# Supplementary material for: Limitations of Linear Dichroism Spectroscopy for Elucidating Structural Issues of Light-Harvesting Aggregates in Chlorosomes
Source: Molecules. 2021 Feb 9;26(4):899. doi: 10.3390/molecules26040899 (PMC7914687; doi:10.3390/molecules26040899)
Supplement: Supplementary file 1 [file molecules-26-00899-s001.pdf]

Supporting Information for:

Limitations of Linear Dichroism Spectroscopy for Elucidating Structural Issues of Light-Harvesting Aggregates in Chlorosomes

Lisa M. Günther [1], Jasper Knoester [2] and Jürgen Köhler [1,3,4,*]

[1] Spectroscopy of Soft Matter, University of Bayreuth, Universitätsstr. 30, 95440 Bayreuth, Germany; lisa.guenther@uni-bayreuth.de
[2] University of Groningen, Zernike Institute for Advanced Materials, Nijenborgh 4, 9747 AG, Groningen, The Netherlands; j.knoester@rug.nl
[3] Bayreuth Institute for Macromolecular Research (BIMF), University of Bayreuth, Bayreuth 95440, Germany
[4] Bavarian Polymer Institute, University of Bayreuth, Universitätsstr. 30, 95440 Bayreuth, Germany
* Correspondence: juergen.koehler@uni-bayreuth.de

Contents:

1. Comparison of reconstruction methods 1 and 2
2. Comment on observing nodes in the LD spectra

1. Comparison of LD Reconstruction Methods 1 and 2

For comparing the two methods used to reconstruct the LD spectra, for simplicity we consider only two transitions with spectra $A_1(\nu)$ and $A_2(\nu)$. We first assume that the two transitions are associated with mutually orthogonal transition-dipole moments, both oriented perpendicular with respect to the incidence direction of light. This is the case for perfectly cylindrical aggregates with their axes parallel to the experimental substrate. For definiteness, we identify transition 1 with the direction parallel to the axis of the cylinder. Then the intensity of the polarization resolved spectrum corresponds to

$$I(\nu, \alpha) = A_1(\nu)\cos^2\alpha + A_2(\nu)\sin^2\alpha \tag{1}$$

where α denotes the angle of the polarization of the light and α = 0 has been chosen to refer to the orientation of the transition-dipole moment associated with transition 1. The polarization-averaged spectrum is thus given by

$$I(\nu) = \frac{1}{2}\big(A_1(\nu) + A_2(\nu)\big) \tag{2}$$

For method 1 we selected the photon frequency ν_{peak} where $I(\nu)$ features its spectral peak and defined the preferential alignment direction as the angle $\alpha_{\|}$ for which the modulation of $I(\nu_{\text{peak}}, \alpha)$ reaches its maximum as a function of α. Accordingly, $\alpha_{\|}$ is found from

$$\frac{d}{d\alpha} I(\nu_{\text{peak}}, \alpha) = 0 \text{ (at } \alpha = \alpha_{\|}) \tag{3}$$

which yields

$$-2\left(A_1\left(\nu_{\text{peak}}\right) - A_2\left(\nu_{\text{peak}}\right)\right)\cos\alpha_{\|}\sin\alpha_{\|} = -\left(A_1\left(\nu_{\text{peak}}\right) - A_2\left(\nu_{\text{peak}}\right)\right)\sin 2\alpha_{\|} = 0 \tag{4}$$

Assuming that $A_1(\nu_{\text{peak}}) \neq A_2(\nu_{\text{peak}})$, this is fulfilled for $\alpha_{\|}$ = 0, which is the polarization angle at which a maximum is found if $A_1(\nu_{\text{peak}}) > A_2(\nu_{\text{peak}})$, and for $\alpha_{\|}$ = π/2, which gives the maximum if $A_1(\nu_{\text{peak}}) < A_2(\nu_{\text{peak}})$. Hence, for exactly perpendicular transition-dipole moments, method 1 selects for the preferential alignment direction either $\alpha_{\|}$ = 0, which agrees exactly with method 2 [where this direction was chosen along the cylinder axis], or $\alpha_{\|}$ = π/2, which is out of phase relative to method 2 by π/2, resulting in a sign flip for the LD spectrum obtained from method 1 with respect to the spectrum obtained from method 2.

This shows that for a system with two transitions with exactly perpendicular transition dipoles, methods 1 and 2 lead to identical single-system LD spectra, up to a possible overall sign change. By direct extension, the above also holds if we have two pairs of transitions [(1,2) and (3,4)], where within each pair the transition dipoles are exactly perpendicular to each other, while 1 is exactly parallel to 3 and 2 is parallel to 4.

However, if the transitions do not have pairwise perpendicular dipoles, but rather have dipole orientations that differ by $\frac{\pi}{2} + \beta$ with $\beta \neq 0$, the above no longer holds. We then have for the case of two transitions:

$$I(\nu, \alpha) = A_1(\nu) \cos^2 \alpha + A_2(\nu) \sin^2(\alpha + \beta) \tag{5}$$

which yields the same polarization averaged spectrum $I(\nu)$ as above. The angle $\alpha_{\parallel}$ for which the modulation of $I(\nu_{\text{peak}}, \alpha)$ reaches its maximum as a function of α now obeys the equation

$$A_1\left(\nu_{\text{peak}}\right) \sin 2\alpha_{\parallel} - A_2\left(\nu_{\text{peak}}\right) \sin 2(\alpha_{\parallel} + \beta) = 0 \tag{6}$$

In general, the solutions for $\alpha_{\parallel}$ will no longer be given by 0 and π/2; rather, their numerical values will depend on both the ratio of the intensities at the peak frequency, $A_1(\nu_{\text{peak}})/A_2(\nu_{\text{peak}})$), and the "mismatch angle" β of their associated transition-dipole moments from being orthogonal. Hence, for β ≠ 0 the results for the LD spectrum obtained from method 1 and method 2 are not equivalent to each other.

The above is nicely illustrated in Fig. S1, which shows in addition to Fig. 5 of the main text also the underlying decomposition of the single-chlorosome spectra in four Gaussians and gives for each case the corresponding angles between the dipoles of the four transitions [1,2]. As is seen, indeed the agreement between both methods used to reconstruct the LD spectrum is better if for both pairs these angles get closer to π/2.

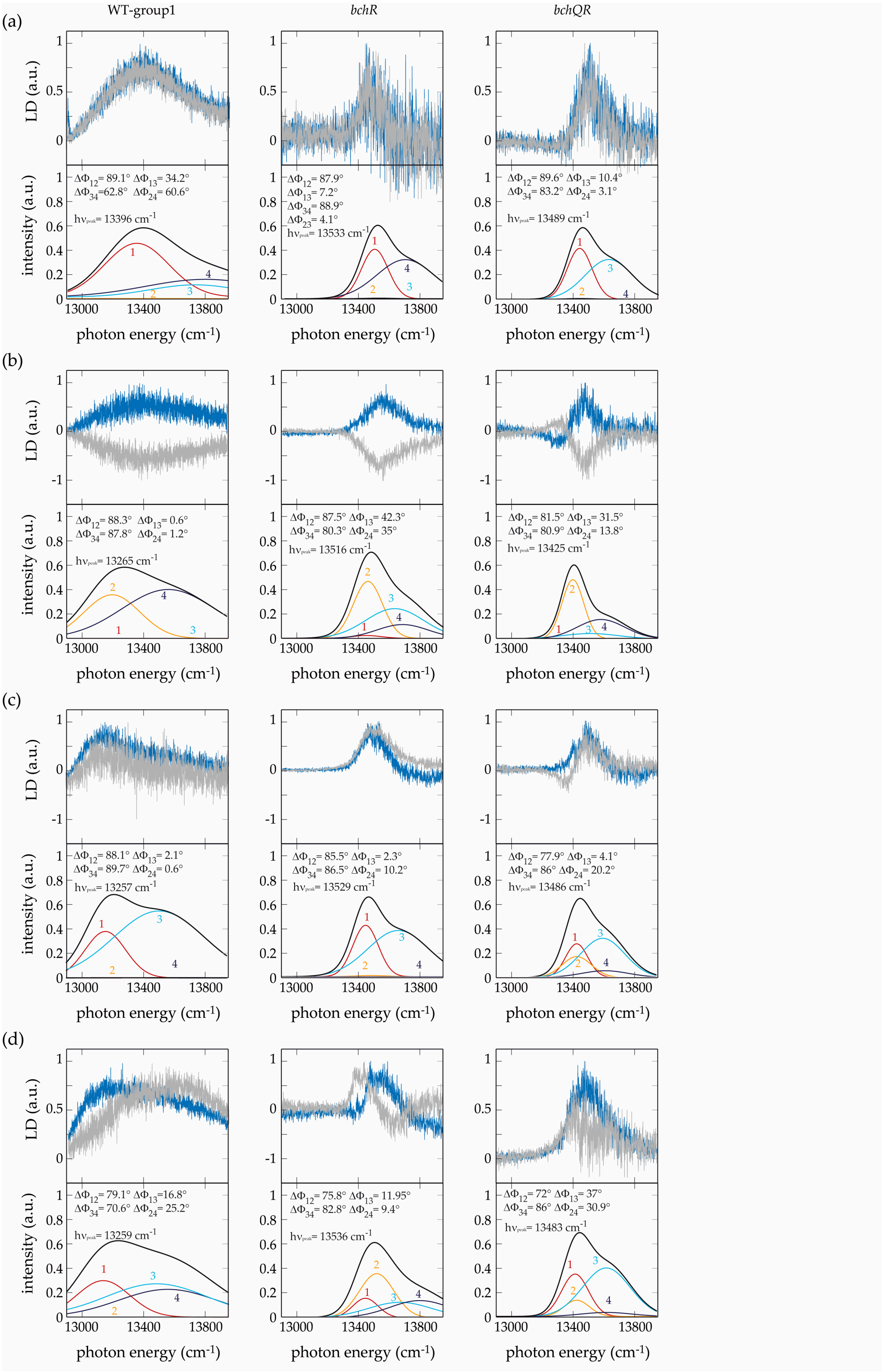

Figure S1. Examples of LD spectra and snapshots of the decomposition into Gaussians at $\alpha_{\parallel}$ for chlorosomes of the WT-group1, the *bchR* mutant, and the *bchQR* mutant, from left to right. The spectra have been obtained from method 1 (blue) and method 2 (grey), respectively. The phase differences $\Delta\Phi_{ij}$ between the bands *i* and *j* (*i,j* = 1,2,3,4), and the energy of the intensity maximum, $h\nu_{peak}$, are given in the panels. (**a**) Group A (good agreement), (**b**) Group B (good agreement but a sign flip), (**c**) Group C (reasonable agreement), (**d**) Group D (no agreement). A - D refer to the four groups used in table 1 of the main text.

For illustration purposes we will discuss two example spectra that were assigned to groups A and D, respectively, in more detail. For doing so we choose the spectra at the top right and the bottom right of fig.S1.

Spectrum fig.S1a, right (group A): The photon energy $h\nu_{\text{peak}}$ where $I(\nu)$ features its spectral peak corresponds to the maximum of the black line of the lower part of this figure. At this frequency this spectrum has dominant contributions from A_1 and A_3. The mutual phase differences amount to $\Delta\Phi_{12}$ = 89.6°, $\Delta\Phi_{34}$ = 83.2°, $\Delta\Phi_{13}$ = 10.4° and $\Delta\Phi_{24}$ = 3.1°. These phase differences are close to the "ideal" situation of having mutually orthogonal transition-dipole moments for A_1 and A_2 (and for A_3 and A_4) as well as pairwise parallel transition-dipole moments for A_1 and A_3 (and for A_2 and A_4). Hence, at $h\nu_{\text{peak}}$ the modulation of the total signal as a function of the polarization and the modulation of the contribution from A_1 are similar and the results from the two reconstruction methods are in accord with each other.

Spectrum fig.S1d, right (group D): At the photon energy $h\nu_{\text{peak}}$ where $I(\nu)$ features its spectral peak this spectrum has contributions from A_1, A_2, and A_3. For the corresponding phase differences we find $\Delta\Phi_{12}$ = 72°, $\Delta\Phi_{34}$ = 86°, $\Delta\Phi_{13}$ = 37°, and $\Delta\Phi_{24}$ = 30.9°. Hence, the mutual phase differences deviate clearly from the "ideal" situation and therefore the modulation of the total signal as a function of the polarization at $h\nu_{\text{peak}}$ differs significantly from the modulation of A_1. As a consequence of this, the two reconstruction methods will give different results.

To summarize: For (close to) "ideal" polarization properties the two reconstruction methods give similar results. A sign flip between the reconstructed LD spectra is observed if at the peak frequency the sum of the contributions from A_2 and A_4 is larger than the sum of the contributions from A_1 and A_3. For strong deviations from the "ideal" geometry of the mutual alignment of the transition dipole moments significantly different LD spectra will be obtained from the two reconstruction methods

2. Comment on Observing Nodes in the LD Spectra

If we assume the four Gaussians to have "ideal" polarization properties we would expect to observe three nodes in the LD spectra. Whether these can be resolved depends on the spectral separation between the states within each pair, the spectral separation between the pairs, the relative intensity of the individual bands, and the widths of all these bands. This is illustrated in fig.S2 on the example of spectra from individual chlorosomes from the *bchR* mutant.

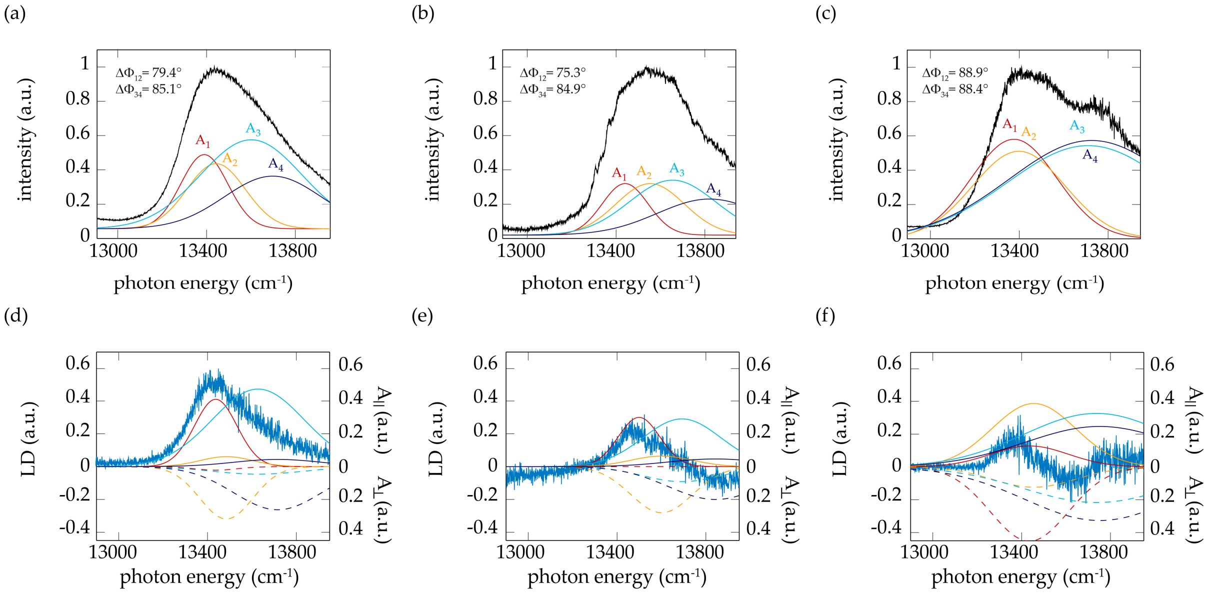

Figure S2: (**a**)–(**c**) (top row): Fluorescence-excitation spectra of individual chlorosomes from the *bchR* mutant averaged over all polarizations (black) together with their decompositions into four Gaussians, A_1 ... A_4, shown in yellow, red, cyan and dark blue. (**d**)–(**f**) Reconstructed LD spectra for the three individual chlorosomes (blue noisy lines) according to method 1. For illustration the contribution from each of the Gaussians to $A_{||}(\nu)$ is plotted along the positive intensity axis (full coloured lines), and the contribution of each of the Gaussians to $A_{\perp}(\nu)$ is plotted along the negative intensity axis (dashed coloured lines).

Concerning the relative abundancies of the nodes across the different types of chlorosomes one has to consider that growing linewidths will wash out the nodes. Since the widths of the bands are significantly smaller for the mutants the nodes can be observed better for these chlorosomes. The variation of the number of nodes is

caused by the variations of the spectral separations and widths of the bands and reflects the (spectral) heterogeneity of the chlorosomes.

References

1. Günther, L.M.; Löhner, A.; Reiher, C.; Kunsel, T.; Jansen, T.L.C.; Tank, M.; Bryant, D.A.; Knoester, J.; Köhler, J. Structural Variations in Chlorosomes from Wild-Type and a bchQR Mutant of Chlorobaculum tepidum Revealed by Single-Molecule Spectroscopy. *J. Phys. Chem. B* **2018**, *122*, 6712–6723, doi:10.1021/acs.jpcb.8b02875.
2. Günther, L.M.; Jendrny, M.; Bloemsma, E.A.; Tank, M.; Oostergetel, G.T.; Bryant, D.A.; Knoester, J.; Köhler, J. Structure of Light-Harvesting Aggregates in Individual Chlorosomes. *J. Phys. Chem. B* **2016**, *120*, 5367–5376, doi:10.1021/acs.jpcb.6b03718.
